# Conducting Online Behavioral Research Using Crowdsourcing Services in Japan

**DOI:** 10.3389/fpsyg.2017.00378

**Published:** 2017-03-14

**Authors:** Yoshimasa Majima, Kaoru Nishiyama, Aki Nishihara, Ryosuke Hata

**Affiliations:** ^1^Department of Psychology for Well-Being, Hokusei Gakuen UniversitySapporo, Japan; ^2^Department of Foreign Language Education, Hokusei Gakuen UniversitySapporo, Japan; ^3^Department of Social Work, Hokusei Gakuen UniversitySapporo, Japan

**Keywords:** online study, non-MTurk crowdsourcing, personality, reasoning, instructional manipulation check

## Abstract

Recent research on human behavior has often collected empirical data from the online labor market, through a process known as crowdsourcing. As well as the United States and the major European countries, there are several crowdsourcing services in Japan. For research purpose, Amazon's Mechanical Turk (MTurk) is the widely used platform among those services. Previous validation studies have shown many commonalities between MTurk workers and participants from traditional samples based on not only personality but also performance on reasoning tasks. The present study aims to extend these findings to non-MTurk (i.e., Japanese) crowdsourcing samples in which workers have different ethnic backgrounds from those of MTurk. We conducted three surveys (*N* = 426, 453, 167, respectively) designed to compare Japanese crowdsourcing workers and university students in terms of their demographics, personality traits, reasoning skills, and attention to instructions. The results generally align with previous studies and suggest that non-MTurk participants are also eligible for behavioral research. Furthermore, small screen devices are found to impair participants' attention to instructions. Several recommendations concerning this sample are presented.

## Introduction

Online survey research is becoming increasingly popular in psychology and other social sciences on human behavior. Researchers often collect data from participants in online labor markets, through a process known as *crowdsourcing*. Recruiting participants from a crowdsourcing service is attractive to researchers because of its advantages over using *traditional* samples.

Estelles-Arolas and Gonzalez-Ladron-De-Guevara (2012) define crowdsourcing as an online activity in which a group of diverse individuals (users) voluntarily undertake a task proposed by an individual or a profit/non-profit organization (crowdsourcer) and in which the users receive monetary and other forms of compensation in exchange for their contributions, while the crowdsourcer benefits from the work performed by the users. In behavioral research, crowdsourcing websites offer researchers a useful platform that provides convenient access to a large set of people who are willing to undertake tasks, including research studies, at a relatively low cost. One of the most well-known crowdsourcing sites is Amazon's Mechanical Turk, which is often abbreviated as MTurk.

MTurk specializes in recruiting users, who are referred to as *workers*, to complete small tasks that are known as *HIT*s (human intelligence tasks). For research purposes, researchers (*requesters*) post a HIT that contain surveys and/or experiments that can be completed on a computer using supplied templates. Sometimes, requesters post a link to external survey tools, such as SurveyMonkey and Qualtrics. Workers will browse or search tasks, and they are paid in exchange for their successful contribution to a task.

Mason and Suri (2012) identified four advantages of MTurk: access to a large, stable pool of participants; participant diversity; a low cost and built-in payment system; and faster theory and/or experiment cycle. Because of these benefits, MTurk is becoming popular as a potential participant pool for psychology and other social sciences.

Along with increasing usage in behavioral research, the validity of the data obtained from MTurk participants has been examined in several studies (for a recent review, see Paolacci and Chandler, 2014). These investigations have typically compared MTurk data with those from traditional samples, such as university students and other community members. First, demographic surveys have shown that MTurk workers are mostly residents of the United States and India and that they are in about their thirties, which is older than typical students who are in their late teens and twenties (e.g., Paolacci et al., 2010; Behrend et al., 2011; Goodman et al., 2013). In addition, workers and participants from traditional samples differ in terms of their personality traits. For example, MTurk workers are less extraverted and emotionally stable and show lower self-esteem than students. They also value money more than time and exhibit higher materialism than an age-matched community sample (Goodman et al., 2013).

The two samples were also different in their performances of reasoning and attention to instructions. For example, Goodman et al. (2013) found that MTurk workers show lower cognitive capabilities than students on the Cognitive Reflection Test (Frederick, 2005), which requires effortful system-2 thinking and on a “trap” task that involves what is known as instructional manipulation checks (IMCs), which reflect participants' inattentive response to the survey questions. However, Goodman et al. also pointed out that failures in IMC were mainly found in ESL and non-US participants. Therefore, the language proficiency, as well as careful reading of study materials, is essential for the successful solution to IMC. Furthermore, Hauser and Schwarz (2015) showed that answering IMCs before “tricky” reasoning tasks improves performance of subsequent tasks; the authors explained that the IMC itself alters participants' attention to subsequent tasks and prompts participants to adopt a more deliberative thinking strategy, which results in improved performance on these tasks.

The MTurk and traditional samples also have several commonalities. For example, MTurk workers and students show similar performance on classical heuristic-bias judgment tasks, such as the *Linda problem* (Tversky and Kahneman, 1983) and the *Asian disease problem* (Tversky and Kahneman, 1981). Paolacci et al. (2010) showed that both students and MTurk workers exhibited a significant framing effect, conjunction fallacy, and outcome bias. They also exhibit a significant anchoring-and-adjustment effect; however, the anchoring bias is mainly shown in the community sample, and the MTurk workers do not show anchoring bias, partly because they might “search” the correct answer on the Internet. In addition, MTurk workers perform similarly in traditional experimental psychology tasks, such as the Stroop, Flanker, attentional blink, and categorical learning tasks (Crump et al., 2013).

In sum, although MTurk participants and traditional participants differ in terms of a few features, they share many common properties. Therefore, crowdsourcing is considered to be a fruitful data collection tool for psychology and other social sciences (Goodman et al., 2013; Paolacci and Chandler, 2014).

MTurk appears to provide a promising approach to behavioral studies owing to its advantages over traditional offline data collection. Despite these advantages, there are some limitations of MTurk as a participant pool for empirical studies. First, there are issues with sample diversity. Demographic surveys have repeatedly shown that the majority of MTurk workers are Caucasian residents of the United States, followed by Asian workers who live in India (Paolacci et al., 2010; Behrend et al., 2011; Goodman et al., 2013). Currently, MTurk requires their workers to provide valid US taxpayer identification information when they get paid (either in US dollars or Indian Rupees), otherwise they can only transfer their earnings to Amazon's gift card. This restriction on monetary compensation may substantially reduce the number of non-US workers. Because of its biased population, it is difficult for researchers in other countries to collect data from residents of their own cultures. Of course, there are other crowdsourcing services, such as Prolific Academic and CrowdFlower, although it seems that Caucasian residents of the USA, the UK, and other European countries are also the predominant participants of these pools. Therefore, researchers who wish to collect data from samples of other ethnicities or nationalities should utilize other crowdsourcing services. This is exactly the case with Japanese researchers.

The second issue is of a technical nature. At this time, a US bank account is required to be a *requester* in MTurk. This requirement also constitutes an obstacle to adopt MTurk as a participant pool for researchers outside the Unites States^1^. On these grounds, MTurk is considered to provide limited access to participant pools for behavioral researchers around the world.

Several studies also pointed out potential pitfalls of online studies with MTurk. First, Zhou and Fishbach (2016) claimed that researchers should pay attention to attrition rate that poses a threat to internal validity of the study. They also recommended that researchers not only implement dropout-reduction strategies, but also explore causes of, increase the visibility of, and report participant attrition. Second, Chandler et al. (2014, 2015) suggested that MTurk workers are likely to participate in multiple surveys, hence workers might be less naïve than participants from other (e.g., student) samples. They also pointed out that the prior experience with commonly used survey question (e.g., Cognitive Reflection Test, Frederick, 2005) inflates performances on the task, and suggested that the repeated participation of workers may threaten the predictive accuracy of the task and reduce effect sizes of research findings. Furthermore, Stewart et al. (2015) estimated the size of the population of active MTurk workers and suggested that the average laboratory can collect data from the relatively smaller numbers of active workers (about 7,300 compared to 500,000 registered MTurk workers). Thus, multiple participations to similar surveys are likely to happen than expected. These pitfalls, the high rate of non-naivety of participants in particular, can be resolved if researchers recruit participants from alternative crowdsourcing services. In addition, conducting online surveys and experiments with multiple crowdsourcing platforms will be beneficial for researchers who look for a more diverse sample.

As noted previously, the quality of data collected from MTurk participants have been verified. It is also shown that the other crowdsourcing pools, such as Clickworker and Prolific Academic, are practical alternatives to MTurk (e.g., Lutz, 2016; Peer et al., 2017). However, data from other crowdsourcing samples, particularly from non-Caucasian samples, have not as yet been fully investigated. To promote research using other crowdsourcing services, we must examine whether the data obtained from other crowdsourcing pools are as reliable as those from MTurk.

## Research objectives and general research method

The primary goal of the present study is to extend existing findings of previous validation studies of MTurk to other non-MTurk crowdsourcing samples. Specifically, we investigated the following questions.

Question 1: Do the demographic properties of workers from the other (i.e., non-MTurk) crowdsourcing samples differ from those of students? If so, how are they different?
Question 2: Do psychometric properties, such as personality traits or those of consumer behavior, differ across non-MTurk workers and students?
Question 3: Is the quality of non-MTurk workers' performance on reasoning and judgment tasks relevant to effortful System-2 thinking in comparison with that of students?
Question 4: How do non-MTurk workers respond to “trap” questions? Are they more (or less) attentive to the instructions for these tasks?

The present study compared crowdsourcing participants with university students in terms of their personality, psychometric properties regarding decision making, and consumer behavior (Survey 1), thinking disposition, reasoning performance, and attention to the study materials (Surveys 2 and 3). In all of the surveys, the crowdsourcing participants were recruited from CrowdWorks (a Japanese crowdsourcing service, which is abbreviated as CW hereafter; https://crowdworks.jp). We adopted CW as a participant pool for the following reasons. Firstly, CW has a growing and sufficiently large pool of registered workers for validation studies (a total of more than 1 million workers as of August 2016). Second, because it offers a user interface that is written in Japanese, the majority of workers are native Japanese speakers, and as a result, it enables data collection from participants of different ethnic groups than MTurk. Third, it offers a similar payment system as MTurk, and it does not charge a commission fee for micro tasks. In addition, it accepts several payment methods, such as bank transfer, credit cards, and PayPal. The student sample was collected from two middle-sized universities that are located in Sapporo, which is a large northern city of Japan. The CW participants received monetary compensation in exchange for their participation in the survey. However, the students received extra course credit or voluntarily participated in the survey.

All of the participants answered web-based questionnaires that were administered by SurveyMonkey (Surveys 1 and 2) or Qualtrics (Survey 3). For the CW sample, we posted a link to the survey site to the CW task. When the participants reached the site, they were presented with general instructions, and they were asked to provide their consent to participate in the survey by clicking an “agree” button. If they agreed to take the survey, the online questionnaires were presented in a designed sequence. After they completed the questionnaires, they received a randomized completion code, and they were asked to enter it into the CW task page to receive payment. Because CW allocates a unique ID per person, it is possible to restrict the same worker to a single task more than once. In addition, we also enabled SurveyMonkey and Qualtrics restriction features to prohibit multiple participations. After the correct completion code had been entered, the experimenter approved the compensation to be sent to the participants' accounts. The CW participants were completely anonymous throughout the entire survey process.

The university students were recruited from introductory psychology, statistics, English, or social welfare classes, and they were provided with a leaflet that described a link to the equivalent web-based survey site. When they reached the site, they received the same general instructions and the same request for their consent to take the survey as the CW participants. After they completed the survey, they were provided with a randomly generated completion code that was required for them to receive credit.

The present study was approved and conducted in compliance with the guidelines of the Hokusei Gakuen University Ethics Committee. All of the participants gave their web-based informed consent instead of written consent.

## Survey 1: personality and psychometric properties

Survey 1 compared the CW and university samples in terms of their demographic status, personality traits and psychometric properties, which included the so-called Big Five traits, as well as self-esteem, goal orientation, and materialism as an aspect of consumer behavior. These scales were adopted from previous validation studies of MTurk (e.g., Behrend et al., 2011; Goodman et al., 2013).

### Method

#### Participants

A total of 319 crowdsourcing workers agreed to participate in the survey; however, we excluded 7 participants because they did not complete the questionnaire. We also excluded 17 responses because of IP address duplication, which left 295 in the final sample. The participants received 50 JPY for completing a 10 min survey.

In addition, we collected 144 students, but we excluded 12 participants from the analyses for the following reasons: incomplete responses (11 participants) and IP address duplication (1 participant). We also excluded one participant from the analyses because of a failure to indicate that he or she was currently a university student in the demographic question. A final sample of 131 undergraduate students participated in the survey.

The sample size of the present survey was decided in reference to previous validation studies of MTurk and other practical reasons. For example, Behrend et al. (2011) collected 270 MTurk and 270 undergraduate students, and Goodman et al. (2013) sampled 207 MTurk and 131 student participants. In addition, based on our previous experience in using CW, we estimated that a growth in the number of CW participants slowed down if we recruited more than 300 participants. Furthermore, the size of the student sample was determined by rather practical reason, i.e., class attendance. However, as shown above, the present survey collected as many student participants as those of Goodman et al. (2013)'s study. Although Goodman et al. (2013) did not mention effect sizes, Behrend et al. (2011) reported that effect sizes on the difference in personality traits between MTurk and student samples ranged from *d* = 0.31–0.86. We conducted power analysis in G-Power to determine sufficient sample size using an alpha of 0.05, a power of 0.8, effect size (*d* = 0.3), and two tails. Based on the aforementioned assumptions, the desired sizes for the first and the second sample were 285 and 127. The result indicated that sample size of the present survey was sufficiently large.

#### Materials and procedure

As the measures for personality traits, we administered two widely used personality inventories: a brief measure of the Big-Five personality dimensions (10-Item Personality Inventory, TIPI; Gosling et al., 2003) and Rosenberg's self-esteem scale (RSE; Rosenberg, 1965). In this survey, we adopted the Japanese version of the TIPI (TIPI-J; Oshio et al., 2012) and the RSE, which was translated into Japanese by Yamamoto et al. (1982). Furthermore, we administered two additional scales that were also used in the previous validation studies: the performance prove/avoid goal orientation scale (PPGO and PAGO; Vandewalle, 1997) and the Material Value Scale (MVS; Richins, 2004). Finally, we asked for participants' demographic status: age, gender, ethnicity, nationality, educational level, and employment status.

All of the participants completed identical measures in an identical order. In the first step, they answered each of TIPI-J items on a 7-point Likert scale (1 = *Disagree Strongly* to 7 = *Agree Strongly*). Next, the participants answered the PAGO and PPGO in mixed order (6-point scale, 1 = *Strongly disagree* to 6 = *Strongly agree*). Subsequently, the participants were presented with 10 items of the RSE followed by a nine-item version of the MVS and provided answers to each item on a 5-point scale that ranged from 1 = *Strongly disagree* to 5 = *Strongly agree*. Finally, they answered demographic questions before the end of the survey.

### Results

All of the statistical analyses of the present study were performed using SPSS 21.0. In addition, when we report η^2^ as an index of effect size of ANOVA, where the value designates partial η^2^.

#### Demographics

Table 1 summarizes the demographic status of both samples. The CW workers were significantly higher in age than the students (UNIV), *M*_C_ = 36.9 vs. *M*_U_ = 19.6 years, *t*_(423)_ = 22.4, *p* < 0.001, *d* = 2.35; percentage of female, CW = 63.7% vs. UNIV = 43.5%, $\chi_{\left(1\right)}^{2}$ = 15.2, *p* < 0.001; and median level of education, Mdn_C_ = “associate degree,” Mdn_U_ = “high school,” Wilcoxon's *Z* = 10.3, *p* < 0.001. The two samples were also different in their years of work experience, *M*_C_ = 12.4 vs. *M*_U_ = 2.5, *t*_(263)_ = 4.7, *p* < 0.001, *d* = 1.17; and employment status, $\chi_{\left(5\right)}^{2}$ = 77.4, *p* < 0.001. On the one hand, 74.8% of the students were not currently employed, and 17.6% were part-time workers. On the other hand, 40.3% of the CW workers were not employed, 21.4% were full-time employees, 19.7% were self-employed, and 13.2% were part-time workers.

**Table 1:** **Demographic results of Survey 1 and 2**.

**Survey**	**Sample**	**Age**		**Female %**	**Work experience (years)**		**Ethnicity**	**Nationality**	
		***M***	***SD***		***M***	***SD***	**%JP**	**%JP**	***N***
1	UNIV	19.56	1.12	43.5	2.47	4.12	98	100	131
	CW	36.89	8.81	63.7	12.38	8.68	98	99	295
2	UNIV	19.70	1.34	46.2	3.04	4.37	98	100	156
	CW	36.59	9.19	62.3	12.43	9.16	98	100	297
			**Middle**	**High**	**Associate**	**Bachelor**	**Graduate**	**NA**	
**HIGHEST EDUCATIONAL LEVEL**									
1	UNIV		0	117	3	10	0	1	131
	CW		1	90	76	118	8	2	295
2	UNIV		0	141	1	9	0	5	156
	CW		9	94	40	114	9	31	297
		**Full-time**	**Part-time**	**Self**	**Employer**	**Retired**	**Not-employed**	**NA**	
**EMPLOYMENT STATUS**									
1	UNIV	0	23	1	1	0	98	8	131
	CW	63	39	58	7	3	119	6	295
2	UNIV	0	26	1	2	0	111	16	156
	CW	69	48	50	5	6	109	10	297

UNIV, student sample; CW, CrowdWorks sample; %JP, percentage of Japanese; Self, self-employed; NA, either “no answer” or “not applicable.”

#### Personality traits

Table 2 summarizes the result of the Big Five personality and self-esteem scale. In the following analyses, we considered sample and gender as independent variables (age was excluded because of a strong point-biserial correlation with sample, *r*_pb_ = 0.74). The gender was included because several previous studies with Japanese participants have shown gender differences in these personality traits (e.g., Kawamoto et al., 2015; Okada et al., 2015; the gender issue was discussed in the Discussion Section). The TIPI-J scores were submitted to a sample × gender MANOVA, and they showed a significant multivariate effect of the sample, *F*_(5, 418)_ = 5.95, Wilk's Λ = 0.93, *p* < 0.001, η^2^ = 0.07. The multivariate effect was also significant for gender, *F*_(5, 418)_ = 6.63, Λ = 0.93, *p* < 0.001, η^2^ = 0.08. The univariate *F*-tests revealed significant differences of the samples in Extraversion, *F*_(1, 422)_ = 8.57, *MSE* = 8.55, *p* = 0.004, η^2^ = 0.02; Agreeableness, *F*_(1, 422)_ = 5.22, *MSE* = 5.38, *p* = 0.023, η^2^ = 0.01; and Conscientiousness, *F*_(1, 422)_ = 6.07, *MSE* = 6.97, *p* = 0.014, η^2^ = 0.01. The two samples were not different in Emotional Stability and Openness (*F*s < 1). The results also showed that males were significantly higher than females in Emotional Stability, *F*_(1, 422)_ = 14.08, *MSE* = 6.39, *p* < 0.001, η^2^ = 0.03; and Openness, *F*_(1, 422)_ = 9.11, *MSE* = 6.34, *p* = 0.003, η^2^ = 0.02. The gender differences were not found in Extraversion, Agreeableness, and Conscientiousness, *F*s_(1, 422)_ < 1.44, *p*s > 0.23. Although the multivariate sample × gender interaction was not significant, *F*_(5, 418)_ = 1.29, Λ = 0.98, *p* = 0.267, η^2^ = 0.02, the univariate analysis showed a significant sample × gender interaction on Openness, *F*_(1, 422)_ = 5.25, *MSE* = 6.34, *p* = 0.022, η^2^ = 0.01. The analysis of the simple main effect indicated that male students were more open than female students, *F*_(1, 422)_ = 10.38, *MSE* = 6.34, *p* = 0.001, η^2^ = 0.02; however, no such difference was found for CW workers, *F* < 1.

**Table 2:** **Means, standard deviations, and Cronbach's alpha coefficients of personality traits and psychometric properties as functions of the sample (UNIV, student; CW, CrowdWorks) and gender (Survey 1)**.

	**UNIV (*****N*** = **131)**							**CW (*****N*** = **295)**						
		**Male (*****n*** = **74)**			**Female (*****n*** = **57)**				**Male (*****n*** = **107)**			**Female (*****n*** = **188)**		
	**α**	***M***	***SD***	**[95% CI]**	***M***	***SD***	**[95% CI]**	**α**	***M***	***SD***	**[95%CI]**	***M***	***SD***	**[95% CI]**
EX^a^	0.706	7.39	3.17	[6.72, 8.06]	7.28	3.30	[6.52, 8.04]	0.746	5.99	2.53	[5.43, 6.55]	6.85	2.91	[6.43, 7.27]
A^a^	0.411	9.82	2.51	[9.29, 10.35]	9.74	2.25	[9.13, 10.34]	0.447	9.09	2.48	[8.65, 9.53]	9.34	2.16	[9.00, 9.67]
C^a^	0.479	6.58	3.03	[5.98, 7.18]	6.26	2.40	[5.58, 6.95]	0.569	6.88	2.31	[6.38, 7.38]	7.36	2.72	[6.98, 7.73]
ES^b^	0.309	7.28	2.61	[6.71, 7.86]	6.04	2.28	[5.38, 6.69]	0.601	7.21	2.29	[6.73, 7.69]	6.43	2.69	[6.06, 6.79]
O^b^^,^ ^c^	0.245	8.50	2.53	[7.92, 9.08]	7.07	2.65	[6.41, 7.73]	0.533	7.81	2.54	[7.33, 8.29]	7.62	2.46	[7.26, 7.98]
RSE^c^	0.814	29.39	7.88	[27.63, 31.15]	26.35	6.14	[24.34, 28.36]	0.889	27.59	7.58	[26.12, 29.05]	28.24	8.13	[27.14, 29.35]
PAGO^a^	0.671	16.31	4.11	[15.56, 17.07]	16.16	2.90	[15.30, 17.02]	0.719	15.19	3.26	[14.56, 15.82]	15.23	3.08	[14.75, 15.70]
PPGO^a^	0.737	16.23	4.28	[15.46, 17.00]	15.42	3.45	[14.54, 16.30]	0.707	14.94	2.97	[14.30, 15.59]	15.02	3.16	[14.54, 15.51]
MVS^a^	0.744	27.97	5.99	[26.63, 29.31]	26.37	6.09	[24.84, 27.89]	0.782	26.16	5.46	[25.05, 27.27]	25.70	5.96	[24.86, 26.54]

*EX, Extraversion; A, Agreeableness; C, Conscientiousness; ES, Emotional Stability; O, Openness to Experience; RSE, Rosenberg's Self-Esteem scale; PAGO, Performance Avoid Goal Orientation; PPGO, Performance Prove Goal Orientation; MVS, Material Value scale*.

a *CW participants are significantly different from students (p < 0.05)*,

b *significant gender difference (p < 0.05)*,

c *significant sample × gender interaction (p < 0.05)*.

A similar ANOVA on the RSE scale failed to show significant sample and gender differences, *F*s_(1, 422)_ < 2.1, *p*s > 0.148. However, we found a significant sample × gender interaction, *F*_(1, 422)_ = 5.02, *MSE* = 59.51, *p* = 0.026, η^2^ = 0.01. The analysis of simple effects revealed that male students scored slightly higher than female students, *F*_(1, 422)_ = 5.0, *p* = 0.026, *MSE* = 59.51, η^2^ = 0.01; however, no gender difference was found for the CW sample, *F* < 1.

#### Goal orientation and material value

Table 2 shows the results of goal orientation and materialism. A MANOVA on two goal orientations indicated the multivariate effect of the sample, *F*_(2, 421)_ = 5.11, Λ = 0.98, *p* = 0.006, η^2^ = 0.02. Subsequent univariate *F*-tests revealed that the students were higher than the workers in both PAGO and PPGO, *F*s_(1, 422)_ = 8.43, 5.45, *MSE*s = 10.93, 11.40, *p*s = 0.004, 0.020, η^2^s = 0.02, 0.01, respectively. However, neither the effect of gender nor the interaction effect were significant, *F*s < 1.

Then, a sample × gender ANOVA was conducted, and the result showed that the students were more materialistic than the crowdsourcing sample, *F*_(1, 422)_ = 3.94, *MSE* = 34.34, *p* = 0.048, η^2^ = 0.01. However, gender main effect and interaction were not significant, *F*s_(1, 422)_ = 2.72, 0.83, *p* = 0.100, 0.362.

### Discussion

In Survey 1, we found a significant, but not surprising, difference between the students and the CW workers in terms of their demographic status. The findings also showed that some personality characteristics differed between the two samples. For example, the CW participants were less extraverted and agreeable, although they were more conscientious than the students. In addition, the CW participants were less materialistic and their pursuit performance-avoid or prove goals were lower than those of the students.

Some of these results, such as demographics, extraversion, openness, and performance-avoid goal orientation, were compatible with the previous validation studies using MTurk (Paolacci et al., 2010; Behrend et al., 2011; Goodman et al., 2013). There were also several inconsistent results on the difference between the two samples compared to the previous studies. For example, Goodman et al. (2013) showed that MTurk workers were more emotionally unstable, i.e., neurotic, than the students and community sample; however, we did not find any such difference between the samples, but we did find a gender difference. Recently, Kawamoto et al. (2015) showed that Japanese females scored higher in neuroticism than males, particularly in their younger adulthood. Our result is compatible with this finding if we consider the distribution of age in both of the samples (UNIV = the majority of the participants were in their late teens or early twenties, CW = 40% were in their thirties, 30% were in their forties, and 19% were in their twenties). Goodman et al. (2013) also found that MTurk workers were less conscientious than students. However, we found an opposite direction of results; our results were consistent with the findings of Big Five personality and showed that conscientiousness was likely to develop during adulthood (e.g., McCrae et al., 2000; Srivastava et al., 2003; Kawamoto et al., 2015). Furthermore, we found that the male students were higher in self-esteem than the female students; however, no gender difference was found in the CW sample. Our findings were compatible with the previous offline investigations, which showed that males had higher self-esteem than females, and this gender difference decreased throughout adulthood (Kling et al., 1999; Robins et al., 2002; Okada et al., 2015).

To summarize, our results indicated both similarities and differences between the CW workers and the students, which is generally consistent with existing findings. It is also important to note that the effect sizes of the sample differences were relatively small, as has been shown in previous studies.

## Survey 2: attentional check and system-2 thinking

Survey 2 aimed to compare the crowdsourcing workers and students in terms of their thinking disposition, as well as their reasoning and judgment biases related to systematic System-2 thinking.

As a measure of thinking disposition, we administered the Cognitive Reflection Test (CRT; Frederick, 2005), which is a set of widely used tasks to measure individual differences in dual process thought, particularly in effortful System 2 thinking. We also administered the following three tasks to measure the participants' biases in reasoning and judgment. The first task was the probabilistic reasoning task (Toplak et al., 2011), which aimed to measure denominator neglect bias in a hypothetical scenario. The second task was the logical reasoning task, which consisted of eight syllogisms (Markovits and Nantel, 1989; Majima, 2015) in which the validity of the conclusion always conflicted with common belief. These syllogisms were designed to measure the strength of the belief bias effect (Evans et al., 1983). The third task was a classical anchoring-and-adjustment task (Tversky and Kahneman, 1974).

We also investigated sample differences in their attention to instructions by using instructional manipulation checks (IMCs; Oppenheimer et al., 2009). In addition, we examined whether answering to the IMCs promoted successful solutions to the other “tricky” reasoning tasks, as shown in Hauser and Schwarz (2015). To investigate whether the interventional effects of an IMC on the subsequent tasks were replicated in the Japanese sample, two questionnaire orders were introduced: IMC-first, in which IMC was administered before the CRT and other reasoning tasks, and IMC-last, in which IMC was administered after those tasks.

### Method

#### Participants

We collected data from 338 CW workers; however, data from 27 of the participants were excluded due to incomplete responses, and data from 14 participants were excluded because of IP address duplication, which left 297 in the final sample. The participants received 80 JPY for the 15 min survey.

We also collected 166 undergraduate students from the same university as in Study 1 as the student sample. However, 10 of the participants were excluded from the analysis for the following reasons: incomplete response = 5 participants, IP address duplication = 1 participant, and failure to choose “student” as the current status at demographic question = 4 participants.

The sample size was decided based on the same rationale as Survey 1. We also conducted power analysis to determine sufficient sample size using an alpha of 0.05, a power of 0.8, effect size (*d* = 0.28), and two tails. The effect size was calculated based on the difference in performance of CRT score between the MTurk and the student participants that was reported by Goodman et al. (2013, Study2). Based on the aforementioned assumptions, the desired sizes for the first and the second sample were 292 and 154. Therefore, the present survey collected the sufficient number of participants.

#### Materials and procedure

In this survey, the participants were presented with five tasks that measured their thinking disposition, reasoning and judgment biases, and attention to instructions: CRT, probabilistic reasoning, syllogism, anchoring-and-adjustment, and IMC. A 2 (sample; CW and UNIV) × 2 (IMC order; first and last) factorial design was adopted.

After the participants read general instructions and provided their consent, those who were assigned to the IMC-first order (*N* = 77 for UNIV, and *N* = 155 for CW) answered IMC questions (sports participation task derived from Oppenheimer et al., 2009). The participants were presented with 11 alternatives that consisted of 10 sports and 1 “other” option, and they were asked to choose activities in which they engaged regularly. However, the instructions also asked the participants to “select other” and enter “I read the instructions” to the text box at the end. If the participants carefully read and followed the instructions, they were scored as “correct.” The participants who were assigned to the IMC-last order (*N* = 79 for student, *N* = 142 for CW) answered IMC questions after the other reasoning tasks.

Next, we administered a three-item version of the CRT (Frederick, 2005). The participants were asked to enter their response into a text entry box. Subsequently, the participants responded to a probabilistic reasoning task (Toplak et al., 2011). In this task, the participants were asked to imagine that they were presented with two trays of black and white *go* stones^2^: a large tray with 100 *go* stones (8 black and 92 white) and a small tray with 10 stones (1 black and 9 white). The participants were also told to imagine that if they drew a black stone, they would win 300 JPY. The participants showed their preference by clicking one of two radio buttons that were labeled “small tray” or “large tray.” The rational choice of this task was the small tray because the chance of winning a prize was higher in the small (1/10) rather than in the large (8/100) tray. However, people often neglect the denominator and prefer the large number of black stones in the large tray.

Then, the participants were presented with the logical reasoning task. They were presented with eight syllogisms one at a time, and they answered by clicking either “True” or “False” on each conclusion. Following the syllogisms, an anchoring-and-adjustment task that was adopted from Goodman et al. (2013) was administered. The participants entered the last two digits of their phone number, they show whether the number of countries in Africa is larger or smaller than that number, and they estimated the exact number of African countries. Finally, we probed whether the participants had previously experienced each of the six tasks. The participants also answered the same demographic questions that were used in Survey 1.

### Results

In the following analysis, the participants who answered “yes” to the probe question to each task were excluded from the analyses.

#### Demographics

Table 1 summarizes the demographic properties of both samples. Similar to Survey 1, the CW participants were significantly different from the students in their age, *M*_C_ = 36.6 vs. *M*_U_ = 19.7, *t*_(451)_ = 22.8, *p* < 0.001, *d* = 2.26; percentage of females, CW = 62.3% vs. UNIV = 46.2%, $\chi_{\left(1\right)}^{2}$ = 10.8, *p* < 0.001, and median level of education, Mdn_C_ = “associate degree,” Mdn_U_ = “high school,” Wilcoxon's *Z* = 9.7, *p* < 0.001. Furthermore, the CW participants had longer work experience than the students, *M*_C_ = 12.4 vs. *M*_U_ = 3.0, *t*_(271)_ = 5.1, *p* < 0.001, *d* = 1.06.

#### IMC performance

Table 3 summarizes the performance of attentional check and the other reasoning tasks. Three of the students and six of the workers were excluded from the following analysis because they answered yes to the probe question. The percentages of the participants who successfully passed the IMC are shown in Table 3. We conducted a logistic regression analysis to ascertain the effects of sample and presentation order on the pass rate of the IMC inquiry. In this analysis, the independent variables were simultaneously introduced into the model. The logistic regression model was statistically significant, $\chi_{\left(3\right)}^{2}$ = 22.83, *p* < 0.001, Negelkerke's pseudo-*R*^2^ = 0.07 (Table 4). The results showed that more CW participants successfully passed the IMC than students, UNIV = 35.3%, CW = 53.6%, odds ratio = 3.44, 95% CI = [1.91, 6.21], $\chi_{\left(1\right)}^{2}$ = 16.8, *p* < 0.001. We also found a significant sample × order interaction, odds ratio = 0.40, 95% CI = [0.18, 0.90], $\chi_{\left(1\right)}^{2}$ = 4.85, *p* = 0.028. Then, we conducted follow-up logistic regression analyses stratified by sample. The order did not affect performance in the student sample; however, the CW participants performed worse if they performed the IMC at the beginning of the survey than at the end of the survey, IMC-first = 45.5%, IMC-last = 62.8%, odds ratio = 0.49, 95% CI = [0.31, 0.79], $\chi_{\left(1\right)}^{2}$ = 8.65, *p* = 0.003.

**Table 3:** **Performance of attentional check and reasoning tasks (Survey 2)**.

		**UNIV (*****N*** = **156)**				**CW (*****N*** = **297)**			
**Task**	**IMC performance**	***n***	***M***	***SD***	**[95% CI]**	***n***	***M***	***SD***	**[95% CI]**
**INSTRUCTIONAL MANIPULATION CHECK % CORRECT^a^^,^^d^**									
IMC first		77	37.7%			154	45.5%		
IMC last		76	32.9%			137	62.8%		
**COGNITIVE REFLECTION TEST^b^^,^^c^**									
IMC first	Pass	22	1.91	1.02	[1.47, 2.35]	62	1.53	1.00	[1.27, 1.80]
	Failure	46	1.17	1.16	[0.87, 1.48]	77	1.09	1.08	[0.85, 1.33]
IMC last	Pass	19	0.84	1.01	[0.36, 1.32]	79	1.48	1.00	[1.25, 1.72]
	Failure	40	1.20	1.07	[0.87, 1.53]	48	0.96	1.11	[0.66, 1.26]
DN % rational^c^	Pass	53	73.6%			154	73.4%		
	Failure	93	64.5%			125	59.2%		
Syllogism^c^	Pass	47	4.49	2.58	[3.73, 5.13]	151	3.51	2.55	[3.14, 3.91]
	Failure	82	3.21	2.08	[2.68, 3.73]	118	3.31	2.37	[2.81, 3.73]
**ANCHORING**									
Mean estimation		147	36.51	17.60		276	38.71	20.26	
*r*^e^			0.171^*^				0.064		

*Sum of ns may not be equal to total number of sample because the number of participants reporting they have experienced the question was different by means of task. UNIV, student sample; CW, CrowdWorks sample; IMC first, IMC was presented before other tasks; IMC last, IMC was presented after other tasks. DN % rational, percentages of participants who chose small, i.e., high probability of win, tray in denominator neglect bias task. IMC order was pooled for results of other reasoning tasks except for CRT*.

a *CW participants are statistically different (p < 0.05) from students*,

b *significant effect of presentation order (p < 0.05)*,

c *significant effect of IMC performance (p < 0.05)*,

d *significant sample × order interaction (p < 0.05)*,

e *Pearson product moment correlation coefficients between the estimated number of African countries and anchor, i.e., last two digits of phone number*.

* *p < 0.05*.

**Table 4:** **Logistic regression analysis predicting the likelihood of passing IMC question by sample and task order, and separate analyses stratified by sample (Survey 2)**.

**Variables**							**Model evaluation**			
	**β**	***SE* (β)**	**Wald's χ^2^**	***p***	**Odds ratio**	**[95% CI]**	**χ^2^**	***df***	***p***	**pseudo-*R*^2^**
[Overall model]							22.83	3	<0.001	0.067
Constant	−0.71	0.24	8.53	0.003	0.49					
Sample (UNIV = 0, CW = 1)	1.24	0.30	16.80	<0.001	3.44	[1.91, 6.21]				
IMC Order (Last = 0, First = 1)	0.21	0.34	0.38	0.537	1.23	[0.63, 2.40]				
Sample × Order	−0.91	0.42	4.85	0.028	0.40	[0.18, 0.90]				
[UNIV]							0.38	1	0.537	0.003
Constant	−0.71	0.24	8.53	0.003	0.49					
IMC Order	0.21	0.34	0.38	0.537	1.23	[0.63, 2.40]				
[CW]							8.80	1	0.003	0.040
Constant	0.52	0.18	8.74	0.003	1.69					
IMC Order	−0.70	0.24	8.65	0.003	0.49	[0.31, 0.79]				

*UNIV, student sample; CW, CrowdWorks sample; pseudo-R^2^, Negelkerke's R^2^*.

#### Cognitive reflection test

We excluded 29 students and 31 workers from the following analysis owing to their previous experience with the task. A 2 (sample) × 2 (IMC order) × 2 (IMC performance; pass vs. failure) ANOVA revealed significant main effects of the IMC performance and task order, *F*s_(1, 385)_ = 7.8, 6.5, *MSE* = 1.12, *p*s = 0.006, 0.011, η^2^s = 0.02, respectively. An order × performance interaction was also significant, *F*_(1, 385)_ = 4.4, *p* = 0.036, η^2^ = 0.01. The analyses of simple main effects revealed that the simple main effect of IMC order was significant among the participants who passed the IMC, *F*_(1, 385)_ = 8.8, *p* = 0.003, *MSE* = 1.12, η^2^ = 0.02; however, the effect of order was not found among those who failed the IMC (*F* < 1). This result indicates that the participants performed better at the CRT only if they successfully solved the IMC question before the CRT. Furthermore, we found a significant three-way interaction, *F*_(1, 385)_ = 5.9, *p* = 0.015, *MSE* = 1.12, η^2^ = 0.02. An analysis of the simple interaction effects showed a significant order × performance interaction for UNIV, *F*_(1, 385)_ = 7.37, *p* = 0.007, η^2^ = 0.02; however, no effect was found for CW (*F* < 1).

#### Denominator neglect and belief bias

The probe analysis excluded 10 students and 18 workers. We conducted logistic regression analyses that predicted the likelihood of high-probability choice by IMC order and performance (see Table 5). The sample was excluded from the model, as the preliminary analysis failed to show any effect of and interactions with the sample. In the first analysis, IMC order, performance, and an order × performance interaction were simultaneously introduced to the model (Model 1), $\chi_{\left(3\right)}^{2}$ = 8.36, *p* = 0.039, pseudo-*R*^2^ = 0.03, AIC = 27.84. However, we failed to find any significant effects of the predictors. Then, we introduced IMC performance solely into the model (Model 2). This model showed a slightly good fit, $\chi_{\left(1\right)}^{2}$ = 6.94, *p* = 0.008, pseudo-*R*^2^ = 0.02, AIC = 15.32. As is shown in Table 5, the participants who successfully passed the IMC tended to choose a higher probability alternative than those who failed at the IMC, 73.4% vs. 61.5%, odds ratio = 1.73, 95% CI = [1.15, 2.61], $\chi_{\left(1\right)}^{2}$ = 6.84, *p* = 0.009.

**Table 5:** **Logistic regression analyses predicting the denominator neglect bias (Survey 2)**.

	**Model 1**					**Model 2**				
**Variables**	**β**	***SE* (β)**	**Wald's χ^2^**	***p***	**Odds ratio [95% CI]**	**β**	***SE* (β)**	**Wald's χ^2^**	***p***	**Odds ratio [95% CI]**
Constant	0.66	0.22	9.23	0.002	1.94	0.47	0.14	11.26	<0.001	1.60
IMC Order (Last = 0, First = 1)	−0.34	0.28	1.40	0.236	0.71 [0.41, 1.25]					
IMC Performance (Failure = 0, Pass = 1)	0.37	0.31	1.42	0.233	1.44 [0.79, 2.63]	0.55	0.21	6.84	0.009	1.73 [1.15, 2.61]
Order × Performance	0.31	0.42	0.55	0.460	1.37 [0.60, 3.14]					
**MODEL EVALUATION**										
χ^2^	8.36					6.94				
*df*	3					1				
*p*	0.039					0.008				
Negelkerke's pseudo-*R*^2^	0.027					0.023				
AIC	27.84					15.32				

*Low probability choice was coded as 0, high probability (rational) choice as 1*.

Next, we conducted a three-way ANOVA on the number of correctly solved syllogisms. In this analysis, 27 students and 28 workers were excluded because of their previous experience with the task. The results showed a significant main effect of IMC performance, *M*_PASSED_ = 3.7 vs. *M*_FAILED_ = 3.3, *F*_(1, 390)_ = 7.6, *p* = 0.006, *MSE* = 5.82, η^2^ = 0.02. We also found marginal main effects of order and sample × performance interaction, *F*s_(1, 390)_ = 2.8, 3.2, *p*s = 0.094, 0.072, η^2^s = 0.01, respectively. *Post-hoc* analyses indicated that the students who successfully passed the IMC scored higher in the syllogism task than those who failed, *F*_(1, 390)_ = 7.7, *p* = 0.006, *MSE* = 5.82, η^2^ = 0.02; on the other hand, the performance of attentional check was not associated with the solution of the syllogisms for the CW workers.

#### Anchoring and adjustment

We excluded 9 students and 18 workers from the following analyses owing to their previous experience. We also excluded three workers who estimated extremely large numbers (>mean +3 *SD*) of African countries, such as 350. To examine the anchoring-and-adjustment effect, we regressed the participants' estimates on their mean-centered phone number (i.e., *anchor*), sample, and an anchor × sample interaction, and we found a marginal positive association between estimates and anchors, β = 0.16, *p* = 0.061; however, this initial model failed to show a good fit, adjusted *R*^2^ = 0.007, *F*_(3, 419)_ = 2.0, *p* = 0.112. Then, we conducted additional separate regression analyses for each of the two samples. On the one hand, we found that the anchor was a significant predictor for the students, β = 0.17, *p* = 0.038, adjusted *R*^2^ = 0.023. On the other hand, this was not the case in the CW sample, β = 0.06, *p* = 0.288, adjusted *R*^2^ < 0.001. These results suggest that students are more prone to the anchoring-and-adjustment bias than CW participants.

The present study allowed the participants to answer the survey using their PC or mobile device at their convenience; therefore, they might have searched for accurate answers on the Internet. Seven students (4.8%) and 26 workers (9.4%) “estimated” the correct number of African countries that could be found on a Wikipedia query (56 countries) or a document by the Ministry of Foreign Affairs of Japan (54 countries). The percentage of correctly “estimated” participants was slightly higher in CW, but the difference was relatively small, $\chi_{\left(1\right)}^{2}$ = 2.89, *p* = 0.089. In addition, the overall percentage of this type of cheating was similar to that found in a previous study (10%; Goodman et al., 2013).

#### Previous experience with the commonly used tasks

The number of excluded participants owing to previous experience was different across tasks. We compared the proportion of participants who answered “Yes” to the probe question between student and CW participants. A series of Chi-square tests revealed that students were more likely to have the experience of participating CRT (% of excluded, UNIV = 18.6 vs. CW = 10.4) and syllogism task (UNIV = 17.3% vs. CW = 9.4%), χ^2^s_(1)_ = 5.92, 5.95, *p*s < 0.02, respectively. However, the proportion of excluded participants was not different in IMC, denominator neglect, and anchoring-and-adjustment tasks, χ^2^s < 1.

### Discussion

Survey 2 showed that the workers and students did not differ in their overall performance in the CRT and probabilistic and logical reasoning. In addition, the CW participants were less prone to anchoring-and-adjustment bias than the non-crowdsourced sample (similar results were reported by Goodman et al., 2013), and this may be partly because the CW participants obtained precisely correct responses from Internet searches. These results are generally consistent with the previous validation studies. However, the present results also show that the students seem not to read instructions carefully in comparison with CW participants and that presentation order has a limited impact on the overall performance of IMC. Contrary to Hauser and Schwarz (2015), mere exposure to IMC did little to improve subsequent reasoning tasks that required systematic thinking. A rather successful solution to the trap question was associated with the successful solution of the other reasoning tasks. Notably, the present participants showed poorer performance on the IMC than those in previous research using a MTurk sample. We suspect that this was partly because the majority of the students accessed the survey site using their mobile device (i.e., smartphones and tablets). We discuss this issue in the section on Survey 3.

The number of participants with prior experience in the task was different between two samples only for CRT and syllogism task. Furthermore, the percentages of previously exposed participants were somewhat lower than MTurk workers. For example, Chandler et al. (2014) indicated that the proportion of workers who reported participating in commonly used paradigms, such as Trolley problem and Prisoner's dilemma was ranged from approximately 10 to 60% (Trolley problem = 30%, Prisoner's dilemma = 56%) except for Dictator's game (0%). The percentages of prior exposure among the present online participants were ranged from 2.0% (IMC) to 10.4% (CRT). Therefore, CW participants seem to be more naïve compared with MTurk workers.

## Survey 3: effect of device type on attentional checks

Survey 2 indicated that the participants were less attentive to the instructions of the task. We suspected that this may have partly been caused by the fact that many of the participants, particularly the students, reached the survey site using small screen devices, such as smartphones. However, because we did not collect information regarding device or browser type in Survey 2, whether small screen devices compared to larger screen devices lead to less attentive responses remains unclear. In Survey 3, we examined whether the use of small screen devices facilitated failure in attentional checks and poorer performance on the other reasoning tasks. As in Survey 2, we also investigated whether the order of the IMC question affected performance in the subsequent reasoning tasks.

### Method

#### Participants

Similar to Surveys 1 and 2, we recruited participants from CrowdWorks; however, in this survey, we decided to hide the task from the workers if their acceptance rate was less than 95%. We collected 205 participants from CrowdWorks; however, 38 of the participants were excluded from analysis for the following reasons: providing incomplete response (3 participants), searching for correct answers or responding randomly (see Materials and Procedure Section; 34 participants), and participating both in mobile and PC surveys (1 participant). One participant in the mobile condition was also excluded because the device type information indicated that he or she had participated in the survey using a PC. Consequently, 167 participants remained in the final sample. (Mobile group, *N* = 81, mean age = 33.4, female = 66.7%; PC group, *N* = 85, mean age = 37.1, female = 44.7%). The participants received 80 JPY for their participation in the survey.

#### Materials and procedure

The tasks and the procedure were almost identical to those of Survey 2 except that the anchoring-and-adjustment task was omitted from this survey. We posted two different CW tasks that were designed for two experimental conditions (device type: Mobile and PC). The participants were asked to choose one of two tasks appropriate for their device. And they were also asked not to participate twice. Both of the tasks consisted of the same general instructions, and the link to the online survey was administered by Qualtrics. The two tasks differed in terms of the following device-specific instructions. The instructions for the mobile condition asked the participants to use their mobile devices (smartphone or tablet) and not to use a PC. However, the participants in the PC condition were asked to take the survey using their PC. In addition, we collected device-type information (e.g., OS, Browser and its version, and screen resolution; these data were collected by Meta Info question of Qualtrics) to prevent those who accessed with inappropriate devices from participating in the survey. At the end of the survey, the participants were presented with two probe questions that asked whether they searched for any correct answers or responded randomly during the survey. Those who answered yes to at least one probe question were excluded from the following analyses.

### Results and discussion

#### Demographics

Table 6 shows the demographic status and performance of the four tasks. The participants in the mobile group were younger than those in the PC group, *t*_(164)_ = 2.6, *p* = 0.009, *d* = 0.41; and the percentage of females was higher in the mobile group than in the PC group, $\chi_{\left(1\right)}^{2}$ = 8.1, *p* = 0.004.

**Table 6:** **Demographic results and performance of reasoning tasks as a function of device type and IMC performance (Survey 3)**.

**Variable**		**Mobile (*****N*** = **81)**				**PC (*****N*** = **85)**			
		***n***	***M***	***SD***	**[95% CI]**	***n***	***M***	***SD***	**[95% CI]**
Age^a^			33.4	8.99			37.1	9.07	
Female %^a^			66.7%				44.7%		
% Passed IMC^a^	IMC first	44	72.7%			40	92.5%		
	IMC last	37	64.9%			45	93.3%		
CRT	Passed IMC	52	1.54	1.11	[1.24, 1.83]	64	1.63	1.02	[1.35, 1.88]
	Failed IMC	21	1.05	1.07	[0.59, 1.51]	6	1.17	1.17	[0.31, 2.03]
DN % rational^b^	Passed IMC	54	64.8%			70	78.6%		
	Failed IMC	24	62.5%			6	66.7%		
Syllogism	Passed IMC	56	4.34	1.94	[3.73, 4.94]	79	4.32	2.40	[3.79, 4.80]
	Failed IMC	25	4.12	2.55	[3.23, 5.03]	6	4.33	1.63	[2.50, 6.16]

*Sum of ns may not be equal to total number of sample because the number of participants reporting they have experienced the question was different by means of task. IMC first, IMC question was administered at the beginning; IMC last, IMC question were administered after other tasks. DN % rational, percentages of participants who choosing the high probability of win option in denominator neglect bias task. IMC order was pooled for results of the other reasoning tasks*.

a *mobile participants are significantly different from PC participants*,

b *mobile participants are marginally different from PC*.

#### Attentional check

The likelihood of passing IMC instructions is shown in Table 6. We conducted a logistic regression analysis to ascertain the effects of device type and task order on the IMC solution, and we found that the logistic regression model was statistically significant, $\chi_{\left(3\right)}^{2}$ = 17.0, *p* < 0.001, pseudo-*R*^2^ = 0.16. The participants in the mobile group were less attentive than the participants in the PC group, 69.1 vs. 92.9%, odds ratio = 0.22, 95% CI = [0.06, 0.83], $\chi_{\left(1\right)}^{2}$ = 4.94, *p* = 0.026; however, presentation order did not affect performance, first = 82.1% vs. last = 80.5%, odds ratio = 1.14, $\chi_{\left(1\right)}^{2}$ = 0.02, *p* = 0.881. Similarly, the device × order interaction was not significant, odds ratio = 0.61, $\chi_{\left(1\right)}^{2}$ = 0.26, *p* = 0.612.

#### Systematic reasoning tasks

A three-way (device type × IMC order × IMC performance) ANOVA on CRT score was conducted; however, 24 participants (8 in mobile 16 in PC group) were excluded from the analysis because they declared that they had experienced with the CRT before the survey. The results showed a marginally significant effect of IMC performance, *M*_PASSED_IMC_ = 1.58 vs. *M*_FAILED_IMC_ = 1.11, *F*_(1, 135)_ = 3.14, *p* = 0.079, *MSE* = 1.13, η^2^ = 0.02; however, none of the other effects were significant, *F*s_(1, 135)_ < 2.2, *p*s > 0.14.

Then, we conducted a logistic regression analysis to predict the likelihood of high-probability choice in a probabilistic reasoning task by device type, IMC order and IMC performance. Three participants in the mobile condition and nine participants in the PC condition were excluded from the following analysis due to previous experience with the task. However, this model failed to show a good fit, $\chi_{\left(7\right)}^{2}$ = 4.77, *p* = 0.688, pseudo-*R*^2^ = 0.04. Instead, the model including device type solely (odds ratio = 0.51, 95% CI = [0.25, 1.05]) showed a slightly good fit, $\chi_{\left(1\right)}^{2}$ = 3.43, *p* = 0.064, pseudo-*R*^2^ = 0.03. This result implies that the participants in the mobile condition tended to neglect the denominator.

Finally, the number of correct responses to eight syllogism tasks was submitted to a similar three-way ANOVA; however, neither the main effects for device type, IMC order, IMC performance nor their interactions were significant, *F*s_(1, 158)_ < 1.3, *p*s > 0.256.

To summarize, Survey 3 indicated that the participants were less attentive to instructions when they used their mobile devices, i.e., small screen devices. They were also prone to denominator neglect bias. However, type of device does not affect other reasoning tasks that are associated with analytic System 2 thinking. Furthermore, the participants were likely to answer reflectively if they read the instructions carefully. These results indicate that small screen devices hinder the careful reading of instructions; however, this might not necessarily spoil the performance of reasoning tasks.

## General discussion

### The characteristics of crowdworks as a participant pool

In the present study, we compared participants from a Japanese crowdsourcing service with a Japanese student sample in terms of their demography, personality traits, reasoning skills, and attention to instructions. In general, the results were compatible with the existing findings of MTurk validation studies. The present results showed many similarities between the CW workers and the students; however, we also found interesting differences between the two samples.

First, but not surprisingly, the CW workers were older and hence had longer work experience than the students. Second, the CW workers and students were different in some of the personality traits, such as extraversion, conscientiousness, and performance-avoid goal orientation; however, these differences were relatively small and compatible with previous MTurk validation studies (Paolacci et al., 2010; Behrend et al., 2011; Goodman et al., 2013) and other studies on personality (e.g., Kling et al., 1999; Srivastava et al., 2003; Kawamoto et al., 2015; Okada et al., 2015). Third, the CW participants performed better at attentional checks; however, they showed similar responses in the other reasoning tasks. These findings suggest that the Japanese crowdsourcing sample was as reliable a pool as that which included MTurk workers.

We also identified a few important dimensions that differed from previous validation studies. First, the present participants, particularly the students, showed poorer IMC performance than participants in previous studies. The failure rate of the present CW participants (46%) was equal to the failure rate in Oppenheimer et al. (2009, Study 1); however, it was remarkably higher than that for recent MTurk workers (e.g., Hauser and Schwarz, 2015, 2016). This might be partly due to the device that was used by the participants to answer the survey. If the participants reached the site using a mobile device (i.e., small screen), they were more likely to miss important instructions than those who used larger screen devices. Second, previous exposure to IMC had a limited impact on the improvement of subsequent tricky-seeming tasks. Hauser and Schwarz (2015) found that answering IMCs prior to a task improved performance on both the CRT and probability reasoning; however, the present results indicated that correctly passing an attentional check, rather than IMC presentation order, was associated with better performance on the other reasoning tasks. Poor performance on the IMC, particularly for the participants who accessed the site with their mobile devices, raises an important methodological issue regarding online data collection. Recently, the penetration rate of smartphone user over population has continued to grow worldwide (e.g., eMarketer, 2014). Moreover, smartphone penetration is much stronger in the younger than the older population. Our findings give the following suggestions. First, IMC could be a useful tool for the elimination of inattentive responses in online studies. Second, it might be wise to ask workers to participate in online studies using relatively large screen devices or to prohibit mobile users from taking the survey. On the other hand, the present findings indicated that IMC performance is moderately associated with other reasoning tasks such as CRT, however the prior exposure to IMC does not necessarily improve performance of subsequent task. Therefore, as one reviewer pointed out, IMC itself might reflect certain personality traits, such as conscientiousness or thoughtfulness, rather than an indicator of a tendency to respond dishonestly. Further, studies would be needed to explore what performances of IMC and other tasks assess.

### Recommendations regarding the use of a non-MTurk participant pool

It is important to note that the present study showed both commonalities and differences between CW workers and the Japanese student sample, which was compatible with the existing literature comprising MTurk validation studies. Despite a few inconsistencies, the present study suggested that online data collection using non-MTurk crowdsourcing services remains a promising approach for behavioral research.

However, at the same time, we recommend that researchers consider the following issues if they collect empirical data from non-MTurk crowdsourcing studies. First, the language that is used in CrowdWorks is limited to Japanese. Therefore, a solid level of language skill is required for both the researchers and the participants to conduct or participate in online surveys with this platform. It may be an obstacle for researchers who are not literate in the Japanese language, and this may also be the case for other crowdsourcing services in which the majority of potential workers are not literate in English. However, in other words, it is also a good opportunity to encourage researchers with different cultural backgrounds to conduct cooperative studies.

Second, MTurk provides a useful command-line interface and API that are designed to control HITs including the ability to obtain a worker's ID. Conversely, CrowdWorks provides only a web-based graphical interface to requesters. This may not necessarily be a disadvantage, since researchers can download the data that includes workers' ID and the survey completion code entered by individual workers from the CrowdWorks website. Therefore, if researchers allocate the unique completion code to each participant, they can examine whether a certain participant has participated in their own surveys before when the naivety in sample is essential. However, it is still impossible to identify whether a certain participant already took similar surveys or experiments that have been administered by other researchers. If the survey includes widely used tasks, such as the CRT, it is helpful to ask participants whether they have already answered previous versions of such tasks. As suggested by several previous studies (e.g., Chandler et al., 2014, 2015; Stewart et al., 2015), data from nonnaïve online participants may threaten the quality of data. Although the present study suggested that the CrowdWorks workers are more naïve compared to MTurk workers for now, a growing usage of this sample will lead some active workers to be *professional* participants, as were MTurk workers. Future investigations should explore whether and how multiple participation across similar surveys might endanger the quality of data from CrowdWorks (and other crowdsourcing) participants.

Third, MTurk workers sometimes receive very little compensation to complete HITs (e.g., $.10 for a 5 min survey) compared to that received in traditional laboratory research (for recent ethical questions concerning online studies, see Gleibs, 2016). In this study, we paid 50 or 80 JPY (approximately 0.40 to 0.70$) for 10–15 min surveys. This rate was relatively higher than that for a typical MTurk study but was still less than the lowest wage of paid workers in the local city (764JPY per hour). It has been shown that data quality does not seem to be impaired by the amount of payment (e.g., Buhrmester et al., 2011; Paolacci and Chandler, 2014); however, the research community may be called on to establish guidelines for ethically valid compensation for participation in surveys.

## Author contributions

YM designed the study, lead the data collection and analysis, and was a main author. KN co-lead the design, assisted in the analysis and interpretation, and was a contributing author. AN assisted in the data collection and contributed with the manuscript drafting. RH co-lead the data collection and assisted in the design of the study.

### Conflict of interest statement

The authors declare that the research was conducted in the absence of any commercial or financial relationships that could be construed as a potential conflict of interest.

## Footnotes

^1^Non-US researchers can post HITs on MTurk, if they use outside service.

^2^In Toplak et al. (2011)'s task, black and white marble were used, although we replaced the marble with the stone of the *go* game, a popular board game in East Asia.
